# MEGDEL Syndrome and Its Anesthetic Implications

**DOI:** 10.7759/cureus.17761

**Published:** 2021-09-06

**Authors:** Balazs Horvath, Kathleen M Pfister, Alexis Rupp, Benjamin Kloesel

**Affiliations:** 1 Anesthesiology, University of Minnesota School of Medicine, Minneapolis, USA; 2 Pediatrics and Neonatology, University of Minnesota School of Medicine, Minneapolis, USA; 3 Anesthesiology, M Health Fairview, Minneapolis, USA

**Keywords:** megdel, megd(h)el, aciduria, phospholipid metabolism, mitochondrial disease, pediatric anesthesia

## Abstract

MEGDEL syndrome gains its name for its following features: 3-methylglutaconic aciduria (MEG), deafness (D), encephalopathy (E), Leigh-like syndrome (L). This syndrome is caused by biallelic mutations in the serine active site-containing protein 1 (*SERAC1* ) gene. When these patients present with hepatopathy (H) in addition to the above manifestations the syndrome is labeled as MEGD(H)EL. The pathology of the disease shares features with different types of inborn errors of metabolism. We present the anesthetic management of a neonate who was diagnosed with MEGD(H)EL syndrome and underwent diagnostic magnetic resonance imaging of the brain at 14 days of postnatal age. We describe the epidemiology and important features of this rare disease that are pertinent for the anesthesiologist.

## Introduction

Advancements in medicine lead to the discovery and characterization of novel congenital syndromes. Therefore, anesthesiologists may encounter patients with newly established syndromes that are not known to them.

MEGDEL syndrome [3-methylglutaconic aciduria (MEG), deafness (D), encephalopathy (E), Leigh-like syndrome (L)] is caused by biallelic mutations in the serine active site-containing protein 1 (*SERAC1*) gene. When these patients present with hepatopathy (H) in addition to the above manifestations the syndrome is labeled as MEGD(H)EL. It was first described in 2006 by Wortmann et al. [1]. *SERAC1 *encodes a protein with a serine-lipase domain, essential for the remodeling of phospholipid phosphatidylglycerol and bis(monoacylglycerol)phosphate, which are necessary for mitochondrial function and cholesterol trafficking, respectively [2]. It is characterized by progressive dystonia, spasticity, failure to thrive, deafness, seizures, and loss of- or failure to meet major developmental milestones. About 50% of those with neonatal-onset have hepatic involvement ranging from severely abnormal liver enzymes, direct hyperbilirubinemia, and hyperammonemia to undulating liver dysfunction, though this is usually transient and occurs predominantly during the first year of life [3]. Another form (juvenile-onset) manifests later in life and has a milder course [4].

MEGD[H]EL syndrome occupies an unusual spot within the landscape of inborn errors of metabolism. While it shares features with 3-methylglutaconic aciduria, which belongs to the organic acidurias, the resulting abnormalities from the *SERAC1* gene mutation also result in impairment of oxidative phosphorylation, a pathomechanism similar to mitochondrial disorders. Still, MEGD(H)EL is distinct enough to be considered part of an emerging class of disorders of phospholipid metabolism affecting the cardiolipin pathway [5].

The syndrome has been reported over 100 times in the literature and is estimated to occur in 27 births worldwide per year with a male: female ratio of 1:1.3 [3,4]. While there are increasing reports of juvenile-onset MEGD(H)EL syndrome with a milder phenotype, many infants present in the neonatal period with severe hypoglycemia and a sepsis-like picture with lactic acidemia. Later in infancy, they suffer from progressive neurologic involvement [4]. Treatment is supportive and survival is about 50% into the teenage years overall [3]. The path to diagnosis and supportive treatments may involve a number of procedures requiring anesthesia, including sedated magnetic resonance imaging (MRI), auditory brainstem response testing, gastrostomy tube placement and/or baclofen pump implantation. Furthermore, as neurologic function deteriorates, there is an increased risk for hospitalizations due to respiratory infections that may require sedation for line placement and/or invasive ventilation.

To date, neither clinical trials nor literature in the form of case reports or series exist that describe the perioperative implications of MEDG(H)EL syndrome. On the other hand, experience with anesthetic care for patients with mitochondrial disorders, organic acidurias, and disorders of fatty acid metabolism can provide insights and help devise a plan that proactively addresses potential problems.

## Case presentation

Written informed consent to publish this case report was obtained from the patient’s parents. We describe the anesthetic management of a female neonate with MEGD(HE)L syndrome at 14 days postnatal age. She was born at 39 weeks and 3 days gestational age via cesarean section due to breech presentation and she weighed 3230 grams. Her 1- and 5-minute Apgar scores were 9/9. She was transferred to our institution from another hospital due to refractory hypoglycemia and hypothermia at 36 hours of life. The work-up for hypoglycemia revealed elevated liver enzymes, hyperbilirubinemia, hyperammonemia, lactic acidosis (Table 1), and generalized muscular hypotonia. Subsequently, she was diagnosed with MEGD(H)EL syndrome by genetic testing which showed p.Arg387X and p.Ser498Thr *SERAC1* variants supporting the diagnosis of autosomal-recessive inheritance. Her metabolic derangements were managed successfully (Table 1), and at 14 days of postnatal age, she was scheduled for magnetic resonance imaging (MRI) without contrast and magnetic resonance (MR) spectroscopy of the brain.

**Table 1:** Pertinent Metabolic Parameters The table shows the most relevant metabolic derangements that are characteristic for MEGD(H)EL syndrome at the time of admission and the corrected values at the time of the MRI scan under anesthesia. (* the value reflects the effect of the glucose-containing maintenance intravenous fluid solution; # blood sample was obtained on postnatal day 4.)

Pertinent Metabolic Parameters	Reference Range	2 days postnatal	14 days postnatal
Glucose (mg/dL)	51-99	< 10	93^*^
Lactate (mmol/L)	0.7-2.0	10.5	1.0
Ammonia (mcmol/L)	10-50	204	27
Gamma Glutamyl Transferase (U/L)	0-65	438	527
Growth Differentiation Factor 15 (pg/mL)	<= 750	> 6,000^#^	-

**Figure 1:**
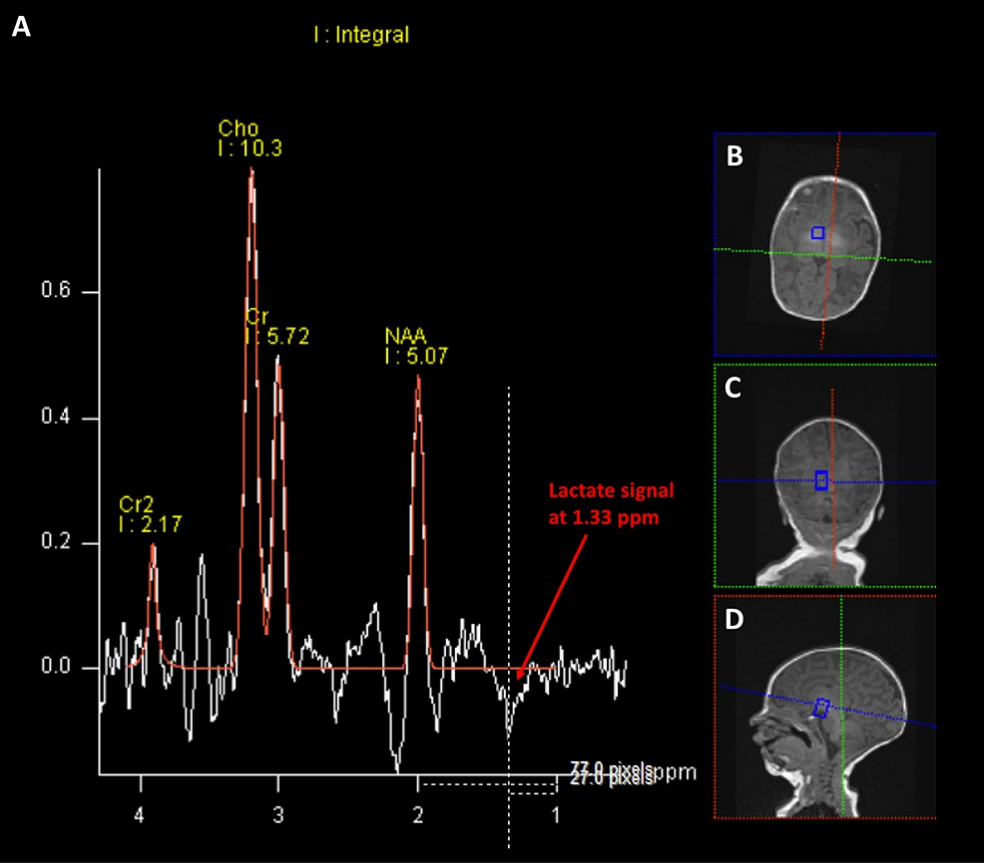
Magnetic Resonance Spectroscopy (A) - Magnetic resonance spectroscopy using intermediate (135 ms) time to echo (TE) sequence. Lactate signal is shown from cubic regions (small box) as an inverted peak at 1.33 ppm. The X-axis depicts the metabolite frequency in ppm according to chemical shift, and Y-axis shows the arbitrary peak amplitude. (B), (C) and (D) - Magnetic resonance imaging in the axial, coronal and sagittal cuts, respectively. Cho = Choline, Cr = Creatinine, Cr2 = Creatinine (second peak), NAA = N-Acetyl Aspartate, ppm = parts per million.

At the time of the diagnostic imaging, her physical exam revealed a right preauricular skin tag, age-appropriate and grossly normal airway anatomy, as well as generalized hypotonia. Because of the underlying congenital metabolic disease, the patient was assigned an American Society of Anesthesiologists Physical Status (ASA PS) score of III. The anesthetic plan was to preserve spontaneous ventilation with a combination of intravenous glycopyrrolate (10 mcg/kg), midazolam (0.15 mg/kg), and dexmedetomidine (1 mcg/kg bolus over 10 minutes followed by 1 mcg/kg/hour continuous infusion). Standard American Society of Anesthesiologists (ASA) monitors, including continuous capnography that is built in the nasal cannula, were used to provide oxygen supplementation. Intravenous fluid replacement and glucose homeostasis were maintained with 5 percent dextrose in 0.2 percent normal saline solution at 4 mL/kg/hour. The anesthetic management was tolerated without complications, and adequate quality images were obtained during the 60-minute-long MRI scan. The MRI showed normal brain structures, and a probable lactate signal was suspected on the intermediate Time to Echo (TE) MR spectroscopy scan (Figure 1). This was not unequivocal as the signal was not a doublet which is usually seen; in addition, there was no convincing upward peak on the long TE images (not shown).

After correction of the metabolic derangement, the patient was discharged home on postnatal day 15. She is being followed in a metabolic clinic and currently takes levocarnitine, vitamin B1, and coenzyme Q-10 with vitamin E. Based on current knowledge, there is no curative treatment for this progressive disease. The infantile-onset type carries a poor prognosis, whereas patients with the juvenile-onset type may survive into the second decade of life, albeit with severe, progressive neurological disabilities [4].

## Discussion

To our knowledge, our case report is the first to describe the anesthetic management of a patient with MEGD(H)EL syndrome, a novel, and rare congenital metabolic disease.

Currently, there is no curative treatment for this progressive disease. The infantile-onset type carries a poor prognosis whereas patients with the juvenile-onset type may survive into the second decade of life, albeit with severe, progressive neurological disabilities [4].

MRI of the brain and MR spectroscopy are valuable diagnostic imaging modalities and were used in our patient in addition to genetic and laboratory testing [4]. In the early phase of the disease, the cerebral structures appear normal. However, MR spectroscopic images can be used to detect the accumulation of neurotoxic metabolites. Lactate, which was suspected on our patient's MR spectroscopy scan, is an important marker of anaerobic metabolism and MR spectroscopy peaks have been previously described in MEGD(H)EL syndrome [4]. They can be detected at 1.33 ppm chemical shift, with a characteristic double peak at long TEs. They are, however, superimposed on the lipid band, and using an intermediate TE will invert only lactate allowing it to be distinguished.

Our proposed anesthetic management of patients with MEGD(H)EL syndrome was driven by the following considerations.

While anatomical features of this syndrome have so far not been associated with difficult airway management, neuromuscular symptoms such as spasticity, dystonia, hypotonia, dysphagia, and excessive drooling can compromise a native airway and may require endotracheal intubation. As in mitochondrial diseases, neuromuscular blockers should be used judiciously and adequate reversal prior to endotracheal extubation is crucial. For patients suffering from seizures, antiepileptic medications should be continued in the perioperative period and may increase resistance to neuromuscular blockers. Patients need to be monitored for malignant arrhythmias. Electrolyte imbalances should be corrected. Cardiac symptoms may include arrhythmias and diastolic dysfunction. Impaired energy production and hypoglycemia should be addressed by minimizing fasting times and by providing glucose-containing intravenous fluids. In complex surgeries that may result in tissue hypoperfusion, fluid resuscitation with lactated Ringer’s may lead to hyperlactatemia and can confuse the clinical picture in the setting of baseline elevated lactate levels.

Regarding mitochondrial dysfunction, it is well-established that many anesthetic agents impair mitochondrial function [6, 7]. The only exceptions are dexmedetomidine with (to date) no known adverse effect on mitochondrial function and ketamine for which conflicting opinions exist [8-10]. Despite this, most agents have been successfully used in those patients when the recommended precautions were followed, however, disease-dependent variable sensitivity to anesthetic agents has been reported [11,12].

We selected the combination of low-dose intravenous midazolam and dexmedetomidine infusion to sedate our patient for the MRI scan. Low-dose benzodiazepines have been shown to be safe in patients with mitochondrial diseases. It appears that dexmedetomidine has no known effects on mitochondrial function [12]. Moreover, dexmedetomidine has been shown to have a mitochondrial protective profile in an animal model [13]. Those factors make it an appealing drug of choice for anesthetic purposes in MEGD(H)EL syndrome and other mitochondrial diseases, especially when no advanced airway device placement is necessary and preserving adequate spontaneous ventilation is desired. Preexisting bradyarrhythmias or heart block, however, may present a relative or absolute contraindication to the administration of dexmedetomidine. While dexmedetomidine is an excellent agent for sedation, it is, by itself, not sufficient for any procedures that require general anesthesia.

It is important to note, that endotracheal intubation should be considered for those patients with the syndrome who are unable to maintain a patent airway or develop apnea or whose underlying metabolic derangements have not been corrected. Other coexisting conditions, such as gastroesophageal reflux and recurrent seizures also warrant endotracheal intubation to prevent pulmonary aspiration, hypoxemia, and hypoventilation. The decision should be based on a risk-benefit analysis. None of those conditions were present in our patient allowing us to avoid potential complications that may arise following endotracheal intubation. Those include airway injury, deferred extubation, or hypoventilation due to the underlying muscular hypotonia, stridor, and subglottic stenosis as a potential delayed complication of endotracheal intubation in neonates.

## Conclusions

As the number of patients who are diagnosed with MEGD(H)EL syndrome is expected to grow, pediatric anesthesiologists should be familiar with the disease. To ensure safe perioperative management, metabolic and phenotypical features of MEGD(H)EL syndrome need to be taken into consideration when designing an anesthetic plan. Selection of anesthetic agents, technique and fluid management should be based on the knowledge of the metabolic derangement and the neurological, neuromuscular and autonomic manifestations. Hypoglycemia is a characteristic symptom of the neonatal onset form and glucose levels should be monitored and stabilized with supplemental glucose-containing infusions.
